# Metagenome-Assembled Genome Sequence of *Rhodopseudomonas palustris* Strain ELI 1980, Commercialized as a Biostimulant

**DOI:** 10.1128/genomeA.00221-17

**Published:** 2017-05-04

**Authors:** Julien Crovadore, Shoutao Xu, Romain Chablais, Bastien Cochard, Delvia Lukito, Gautier Calmin, François Lefort

**Affiliations:** a Plants and Pathogens Group, Research Institute Land Nature and Environment, hepia, HES-SO University of Applied Sciences and Arts Western Switzerland, Jussy, Geneva, Switzerland; b Ecological Laboratories, Inc., Cape Coral, Florida, USA; c Faculty of Engineering and Architecture, HES-SO University of Applied Sciences and Arts Western Switzerland, Delémont, Jura, Switzerland

## Abstract

We report here the draft genome sequence of strain ELI 1980 of *Rhodopseudomonas palustris*, commercialized as a biostimulant for agriculture. The genome was reconstructed from the metagenome of a commercial product containing this strain as its major component.

## GENOME ANNOUNCEMENT

*Rhodopseudomonas palustris* (Molisch 1907) van Niel 1944 (1) is a Gram-negative purple photosynthetic bacterium belonging to the *Bradyrhizobiaceae* family. Rod-shaped and motile, it has been isolated from various environments, including animal waste lagoons (2, 3), sludge (4, 5), aquatic sediments (6), moist leaf litter (7), diverse soils (7–9), rice straw (10), alkaline waters (11), and eutrophicated ponds (3). Because of its capability to function in the four known life metabolism types, photosynthetic, photoheterotrophic, chemoheterotrophic, and chemoautotrophic (12), it has been widely studied for its possible applications in agriculture (3, 9, 13), hydrogen production (14–18), electricity production (5), dehalogenation of carboxylic acids (19), and degradation of aromatic compounds (2, 18, 20, 21). The first genome sequence for this species was published in 2004 (12). Strain ELI 1980 has been isolated from a pond in Suffolk County (NY, USA) and has been used as a component of Quantum Light, a biofertilizer commercialized by Ecological Laboratories, Inc. (FL, USA). Metagenomic DNA was extracted from a 5-ml sample of the biofertilizer, according to a cetyltrimethylammonium bromide (CTAB)-based adapted protocol (22), including RNA digestion with RNase A/T1 (Ambion). Metagenomic DNA was sheared in an AFA microtube (Covaris, USA) in an S2 ultrasonicator (Covaris) to achieve an average fragment size of 350 bp. The sequencing library was created with the TruSeq DNA PCR-free library kit (Illumina, USA). Whole-metagenome shotgun sequencing was carried out within one Illumina HiSeq run at 2 × 125-bp paired-end read length and yielded 37,560,000 reads (4.695 Gb of DNA). Read quality was controlled with FastQC (http://www.bioinformatics.babraham.ac.uk/projects/fastqc). The metagenome assembly was computed with MetaSPAdes metagenomic assembler version 3.10 (23) and aligned to the reference genomes of *R. palustris* strains CGA009 (12) and DX-1 genome (24) using MetaQUAST (25). Matching contigs were extracted into a separate file and arranged with BioEdit (26). The final assembly yielded 21 contigs (≥500 bp), for a total genome length of 5,651,625 bp, a G+C content of 65.05%, and an *N*_50_ value of 327,924 bp. One plasmid (8,482 bp) similar to the plasmid of the strain CGA009 (12) was confirmed by plasmidSPAdes (27). Gene annotations were carried out with the Prokaryotic Genome Annotation Pipeline (PGAP) (28) and RAST version 2.0 (29). PGAP detected 5,177 genes, 5,114 coding sequences (CDSs), 5,052 coding genes, 63 RNA genes (5S, 16S, 23S), tRNAs, and noncoding RNAs (ncRNAs), and 62 pseudogenes, while RAST described 5,280 CDSs spread over 504 subsystems. No toxin genes were identified. The strain is able to produce antibiotics. Genes active in photosynthesis include light-harvesting proteins and a complete photosystem II-type reaction center. Along with a few genes involved in auxin synthesis, this bacterium also fixes atmospheric nitrogen through a complete nitrogenase complex and is well able to nitrify ammonium in nitrates and nitrites. Additionally, it harbors 83 genes active in the degradation of aromatic compounds and is equipped to resist arsenic, cadmium, chromium, cobalt, zinc, and copper. This genetic equipment confirms that this strain could be used in pollution remediation and in agriculture.

### Accession number(s).

This whole-genome shotgun (WGS) project was deposited at DDBJ/EMBL/GenBank under the accession number MVOD00000000. The version described in this paper is the first version, MVOD00000000.1. The 21 contigs have been deposited under the accession numbers MVOD01000001 to MVOD01000021.
